# Investigations of Processing–Induced Structural Changes in Horse Type-I Collagen at Sub and Supramolecular Levels

**DOI:** 10.3389/fbioe.2019.00203

**Published:** 2019-08-26

**Authors:** Alberta Terzi, Nunzia Gallo, Simona Bettini, Teresa Sibillano, Davide Altamura, Lorena Campa, Maria Lucia Natali, Luca Salvatore, Marta Madaghiele, Liberato De Caro, Ludovico Valli, Alessandro Sannino, Cinzia Giannini

**Affiliations:** ^1^Institute of Crystallography (IC), National Research Council, Bari, Italy; ^2^Department of Engineering for Innovation, University of Salento, Lecce, Italy; ^3^Typeone Srl, Lecce, Italy; ^4^Department of Biological and Environmental Sciences and Technologies, University of Salento, Lecce, Italy

**Keywords:** type I collagen, X-rays, FT-IR, biomaterial, structural modification, medical devices, stiffness

## Abstract

The aim of this work is to evaluate the effects of different extraction and material processing protocols on the collagen structure and hierarchical organization of equine tendons. Wide and Small Angle X-ray Scattering investigations on raw powders and thin films revealed that not only the extraction and purification treatments, but also the processing conditions may affect the extent of the protein crystalline domain and induce a nanoscale “shield effect.” This is due to the supramolecular fiber organization, which protects the atomic scale structure from the modifications that occur during fabrication protocols. Moreover, X-ray analyses and Fourier Transform Infrared spectroscopy performed on the biomaterial sheds light on the relationship between processing conditions, triple helical content and the organization in atomic and nanoscale domains. It was found that the mechanical homogenization of the slurry in acidic solution is a treatment that ensures a high content of super-organization of collagen into triple helices and a lower crystalline domain in the material. Finally, mechanical tensile tests were carried out, proving that the acidic solution is the condition which most enhances both mechanical stiffness and supramolecular fiber organization of the films.

## Introduction

Tissue engineering is based on the implantation of biocompatible and biodegradable scaffolds that provides structural support to cell growth and tissue development. The goal is to mimic the microenvironment architecture, mainly represented by the extracellular matrix (ECM), a complex network composed by water and strongly interconnected fibrous/non-fibrous proteins and polysaccharides, that supports tissue growth and mechanical stability and allows optimal homeostasis (Kim et al., 2016). Its biological composition and structural properties are tissue-specific, due to biochemical signals released by different components during tissue morphogenesis and remodeling. Variations of the ECM biological and mechanical structure have been demonstrated not only among different tissues, such as skin and tendon, but also among different areas of the same organ, and different pathologic states (Frantz et al., 2010). The most representative protein of connectives is type I collagen, which amounts to about 70% of total collagen in mammals (Adachi et al., 1997). It is a fibrous protein that confers strength and toughness thanks to its hierarchical supramolecular organization. The molecular structure is composed by two α1 chains and one α2 chain, coiled in a right-handed rod-like triple helix (300 nm long, ~1.1 nm diameter) (Persikov et al., 2004), featuring two non-helicoidal terminal portions named telopeptides, N and C-terms, with 16–26 amino acidic residues. The covalent bonds between amine and carboxyl groups within molecules are mainly located in these regions, stabilizing the molecular arrangement by crosslinking.

The characteristic repetition of Gly-X-Y within each strand preserves the triple helical conformation. The molecular stabilization is also related to the formation of hydrogen bonds in the helical backbone, and to water bridges that, as shown in the literature, support the helical conformation that forms a cylinder of hydration around the collagen crystal lattice (Bella et al., 1995).

Most of the structural features of type I collagen have been assessed and confirmed by X-ray investigations at sub and supramolecular scales. Although studies began in the early 1900s, the first helical model was only proposed in the 1950s by Rich and Crick, and consisted of a triple stranded 10/3 helical model with a 10/1 helical symmetry of each peptide strand, a pitch length of 86 Å and an axial repetition of 28.8 Å with 3.33 residues/turn (Rich and Crick, 1971). Subsequently, Okuyama et al. (2006) detected the 7/2 helical structure in a synthetic single crystal and proposed this structure as a new model for a collagen-like peptide, with a 7/1 helical strand symmetry, a 60 Å pitch length and an axial repetition of 20.0 Å. However, the 7/2 and the 10/3 were both only accepted as possible collagen structure models in 2006. Thus, the essential information about triple helix conformation of collagen comes from Wide Angle X-ray scattering (WAXS) patterns, which were found to be particularly clear in organized tissues such as kangaroo tail tendons.

Collagen molecules are packed in fibril bundles at a molecular distance of about 1.6 nm and assembled in a quarter-staggered order with 64–67 nm periodicity along the central axis of the fibrils, that are in turn packed in fibers with a packing distance of about 100 nm (Brodsky and Ramshaw, 1997; Wilkinson and Hukins, 1999; Shoulders and Raines, 2009).

Small Angle X-ray Scattering (SAXS) has proved to be a suitable technique to investigate the nanoscale architecture of collagen fibers. It has also proved helpful to reveal the alteration in the lateral packing between hydrate and dry tissue. The obtained supramolecular information was used to define a model of collagen association within a compressed quasi hexagonal lateral packing, which has been well-defined by Orgel et al. (2006).

Based on the published literature, X-ray diffraction data also highlight evidence of a significant amount of diffuse scattering within both molecular and fibrillary structures. This has been associated with a dynamic disorder (liquid-like disorder) (Fratzl et al., 1998) which depends on the spatial organization within the tissue; for instance, collagen fibers are mostly oriented longitudinally in tissues subjected to mechanical stress, thus the fibrillary packing is more crystalline than in other soft tissues. In particular, mammalian tendons are mainly composed by hierarchically organized type I collagen, stabilized in a parallel alignment by the surrounding extracellular matrix (ECM) and water content, also termed ground substance (Kannus, 2000). In tendons, specific cells termed tenocytes, are hardwired around collagen fibrils where they secrete ECM components, including other types of collagen, elastin, and proteoglycans (Franchi et al., 2007). The preferred orientation of collagen fibers within this connective tissue facilitates the sliding of fibrils and the transmission of movement (Kahn et al., 2013). In fact, tendons are fibrous connective tissues that bind muscles to bones, transmitting the contraction mechanical force to the skeleton and allowing movement. Due to the abundance of type I collagen, tendons have been considered as the preferential extraction source for industries and the relevant role of collagen hydrogels has been demonstrated in several studies (Che et al., 2006; Gingras et al., 2008). In tissue engineering collagen is used as an anchorage material for tissues and organ regeneration in particular for the treatment of skin diseases, ulcers (Abramo and Viola, 1992), osteochondral defects (Levingstone et al., 2016), vessels (Boccafoschi et al., 2015) and neural regeneration (Di Summa et al., 2014). The great structural variety of collagens, including those extracted by different species and tissues such as bovine dermis, fish skin (Barnes et al., 1982), tendons (Nimni et al., 1987), and jellyfish, is used for the manufacturing of many medical devices. Chemical and enzymatic extraction protocols however deeply affect the collagen sub and supramolecular structure. Chemical extraction commonly disrupts collagen fibers that preserve the native structure of the triple helices. Regarding the enzymatic approach, it is based on the selective cleavage of the terminal non-helical regions (telopeptides) through peptidases (Piez, 1985) that break peptide bonds near the crosslinking sites leading to soluble molecules. The collagen obtained by this approach is called atelocollagen (without telopeptides) and evokes a lower immune response compared to the other one, as telopeptides contain the antigenic P-determinant (Knapp et al., 1977). The resulting extracted collagen possesses poor thermal stability and mechanical strength, however.

Additionally, the manufacturing processes influence the structural properties of type I collagen, as they are carried out using several techniques such as air-drying, freeze-drying, 3D printing, electrospinning and laser treatments. In order to improve the mechanical properties of collagen(s), collagen-based substrates are also subjected to crosslinking methods, including chemical (carbodiimide crosslinker (EDC/NHS), glutaraldehyde, etc.) and physical treatments such as ultraviolet and dehydrothermal treatments (UV, DHT), that can be applied alone or in combination (Sionkowska, 2016). These structural changes may also affect the way in which cells recognize and respond to functional collagen moieties; for instance, DHT crosslinking treatments contribute to the exposure of additional RGD ligands, essential for cell recognition, but they also induce partial collagen denaturation and weakness (Tung et al., 2009).

As shown in our previous study on bovine collagen (Terzi et al., 2018) there is a correlation between different production treatments and collagen structural features at both sub and supramolecular scales, that deeply impact mechanical and biological properties of collagen-based medical devices. In this context, different collagen-based films appear to be good models to investigate the structural changes in protein molecules, as they can be easily modified by physical and chemical treatments. In this work chemical (TYP1CH) and enzymatic (TYP1EN) extraction methods were compared to assess their effects on collagen structural features. Furthermore, the impact of processing protocols commonly used in tissue engineering was investigated to evaluate multilevel modifications of the macromolecule structure and the final properties of the obtained scaffolds.

## Materials and Methods

### Materials

Different commercially available collagen fibers, deriving from equine tendon and extracted through chemical (TYP1CH) and enzymatic protocols (CC, TYP1EN), were analyzed, as specified (Table 1).

**Table 1:** Commercially available collagen fibers for biomedical applications.

	**Enzymatic extraction**	**Chemical extraction**
**COLLAGENS**		
Lyophilized (A)	*TYP1EN_A*	*TYP1CH_A*
Hydrated (B)	*TYP1EN _B*	*TYP1CH_B*

All of the collagen types were provided by Typeone Srl (Lecce, Italy). In particular, TYP1CH_A, TYP1EN_A, TYP1CH_B, and TYP1EN_B were produced by Typeone Srl (Lecce, Italy), according to proprietary protocols, while CC was a commercial collagen source used by Typeone Srl as a control.

All other chemicals used in this work were purchased from Sigma-Aldrich, unless otherwise stated and were used as received.

### Methods

#### Films Preparation

Collagen-based films (about 100 μm thick) were prepared from hydrated and lyophilized raw materials. Hydrated collagens (TYP1CH_B, TYP1EN_B), provided in a 0.5 M acetic acid solution (2% w/v), were used to produce thin films, also called AA, and from solutions that were additionally homogenized (OMO) through an overhead blender (20 min, T = 10°C, 7,000 rpm). Lyophilized collagens (CC_A, TYP1CH_A, TYP1EN_A), partially soluble in acidic solutions (AA), were dispersed (2% w/v) in 0.5 M acetic acid and subsequently processed by OMO treatment. In order to remove air bubbles, all slurries were centrifuged for 10 min at 7,000 rpm at room temperature (21°C) and poured into Petri dishes under the hood, allowing solvent evaporation. Finally, all films were peeled off and crosslinked through a dehydrothermal (DHT) procedure, in a vacuum oven at 121°C and 100 m Torr for 72 h, ensuring the stabilization of the collagen network. Additional non-crosslinked films were made as control samples for structural investigations. All samples were sterilized with dry heat, at 160°C for 2 h in vacuum.

#### Sodium Dodecyl Sulfate-Polyacrylamide Gel Electrophoresis (SDS PAGE)

The SDS PAGE technique was employed to assess the degree of purity of the equine isoforms. The Mini-Protean Tetra Cell (Bio-Rad Laboratories, Inc.) electrophoresis system on the acrylamide gels were used for the analysis. Gels (10% resolving gel, 5% stacking gel) were prepared using acrylamide/bisacrylamide solution with a ratio of 37.5:1. Each sample, before the SDS PAGE, was subjected to a treatment with a reducing solution composed by Laemmli buffer (62.5 mm Tris-HCl pH 6.8, 10% glycerol, 2% SDS, 0.01% bromophenol blue, 5% β-mercaptoethanol) and 2 M Urea, in order to disaggregate collagen fibrils (Falini et al., 2004; Ruozi et al., 2007).

A small amount of each collagen gel (0.3 g) was dissolved in 0.5 ml of reducing solution and heated at 50°C for 1 h. Samples were subsequently centrifuged for 1 min at maximum speed and 0.3 μl of each supernatant was collected and loaded in the electrophoresis wells. The SDS PAGE run was carried out at 70 V for ~10 min in the stacking gel and at 120 V for ~2 h within the resolving gel. The standard marker was provided by Bio-Rad Laboratories. The protein bands pattern of CC collagen, used as the control, provided an example of the degree of sample purity. After electrophoresis, the gel was silver stained and bands intensity was evaluated by ImageJ software.

#### X-Ray Scattering Measurements

Wide and Small Angle X-ray Scattering (WAXS and SAXS) experiments over raw material and collagen-based films was performed at the X-ray Micro Imaging Laboratory (XMI-LAB) (Altamura et al., 2012) which is equipped with a Fr-E+ SuperBright copper anode MicroSource (λ = 0.154 nm, 2,475 W) coupled through a focusing multilayer optics Confocal Max-Flux (CMF 15–105) to a three pinhole camera for X-ray microscopy. WAXS data were collected by an Image Plate (IP) detector (250 × 160 mm^2^, 100 μm effective pixel size) placed at around 10 cm distance from the sample, giving access to a range of scattering vector moduli (q = 4πsinϑ/λ) from 0.3 to 3.5 Å^−1^, corresponding to a 1.8÷21 Å range in the direct space. Data were read by an off-line RAXIA reader. SAXS data were collected using a Triton 20 gas filled proportional counter (1,024 × 1,024 array, 195 μm pixel size) placed at ~2,200 mm distance from the sample, in order to have access to a range of scattering vector moduli from 0.006 to 0.2 Å^−1^, corresponding to a 3–105 nm range in the direct space. Three points per sample were analyzed where WAXS/SAXS data were the average across an area equivalent to the beam size, which is about 0.5 × 0.5 mm^2^. Data were collected for 1 h per each point. WAXS and SAXS data were elaborated by SAXSGUI and SUNBIM (Siliqi et al., 2016) software.

#### Infrared Spectroscopy

Raw fibers and collagen-based films were analyzed by Fourier Transform Infrared (FT-IR) Spectroscopy in Attenuated Total Reflection (ATR) modality. Spectra were obtained using a PerkinElmer Spectrum One IR spectrometer in ATR mode with 4 cm^−1^ resolution and average scans of 64.

#### Mechanical Characterization

Tensile tests were performed on collagen-based films crosslinked by DHT treatment with the aim of verifying the efficacy of the crosslinking method, in relation to different types of collagen and processing protocols. Five rectangular shaped replicates (1 × 3 cm) for each film were stretched. Samples were soaked overnight in PBS at room temperature, before the uniaxial elongation trial by a Zwick-Roell mechanical tester. A Dino-Lite digital microscope was employed to evaluate the thickness of the hydrated sample (about 0.15 to 0.20 mm). The films mounted between the clumps of the tester, were submerged in NaCl 0.9% (w/v) bath chamber. Each sample was initially pre-stretched with a load of 0.01 N and the movement of the crosshead was continued with a speed of 1 mm/min until the samples ruptured (Damink et al., 1995).

Engineering stress and strain were calculated from the load-displacement data. Thus, stress (σ) was determined as the ratio between the force registered by the tester (F) and the initial cross-sectional area (width × thickness) of the gauge section of the sample (A_0_):

(1) $$\begin{array}{l} \sigma =\frac{F}{A_{0}} \end{array}$$

And the strain (ε) as

(2) $$\begin{array}{l} \varepsilon =\frac{\Delta l}{l_{0}} \end{array}$$

where Δ*l* is the measured crosshead displacement and *l*_0_ is the initial length. The elastic modulus (E), also known as Young's modulus, is defined as the slope of the stress/strain curve at small strain and was elaborated within the range between 1 and 4% of the material deformation. The Young's modulus (E) of each film corresponds to the average value of the E value of replicates. Statistical analysis was performed by ANOVA test on the five replicates of each films.

## Results

In Table 2 we report the fabrication protocols performed on the raw materials extracted by chemical (TYP1CH) and enzymatic protocols (CC, TYP1EN) and relative films.

**Table 2:** Collagen-based films obtained from hydrated and lyophilized raw materials through different fabrication protocols.

	**2% (w/v) in 0.5M acetic acid (AA)**	**2% (w/v) in 0.5M acetic acid and homogenization (OMO)**
**COLLAGEN-BASED FILMS**		
Lyophilized (A)	–	*CC_OMO_A*
	–	*TYP1CH_OMO_A*
	–	*TYP1EN_OMO_A*
Hydrated (B)	*TYP1CH_AA_B*	*TYP1CH_OMO_B*
	*TYP1EN_AA_B*	*TYP1EN_OMO_B*

All films have been analyzed before and after the DHT treatment. A detailed description of materials and processing methods is reported in section Materials and Methods.

### SDS PAGE Analysis

**Figure 1:**
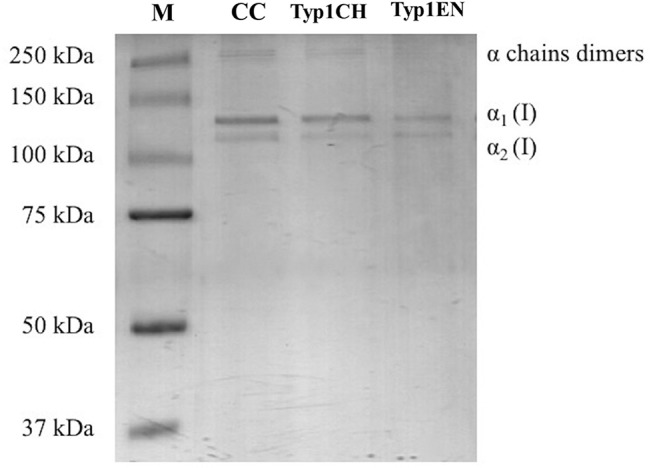
SDS PAGE of collagen suspensions in comparison with the marker M (broad range 37–250 kDa), CC was used as the control for the purity assessment of TYP1CH and TYP1EN.

Sodium dodecyl sulfate polyacrylamide gel electrophoresis demonstrated the purity level of equine collagen isoforms. Figure 1 contains SDS PAGE bands of the weight marker (range 37–250 kDa) and α_1_ and α_2_ chain bands of the samples: collagen control (CC), TYP1CH and TYP1EN types. Data revealed the presence of the typical bands of α1(I) of about 130 kDa and α2(I) chains of about 110 kDa of type I collagen. SDS PAGE of the full-length gel is presented in Supplementary Material.

The quantitative analysis of the proteic bands confirmed the presence of the two α1(I) helices and α2(I) helix, since the intensity of the α1(I) was double compared to the α2(I) band. An additional wide band was also observed in the upper part of the gel, with high molecular weight components that are related to α-chain dimers and trimers (β- and γ- chains). Since no other proteic species were visible, results indicate the purity of collagen TYP1CH and TYP1EN which resulted to be pure at the nanogram. The same purity level was found for CC (Figure 1).

### WAXS/SAXS Analysis

**Figure 2:**
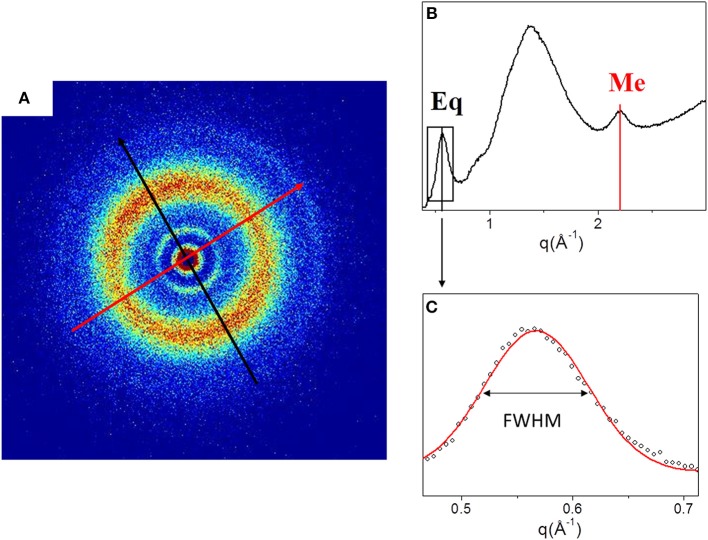
**(A)** Diffracted intensity distribution of TYP1CH_OMO_A; **(B)** Corresponding 1D WAXS profile. **(C)** Gaussian fit elaboration and FWHM calculation of the equatorial diffraction peak.

Figure 2 shows the 2D WAXS pattern (Figure 2A) collected on the TYP1CH_OMO_A film and the corresponding 1D diffraction profile (Figure 2A). The 2D diffraction data (Figure 2A) reveals the presence of two reflections: the meridional (red arrow) which reflects the electron density distribution along the central axis of collagen triple helix, i.e., molecular axial periodicity, and the equatorial (black arrow) which corresponds to the lateral packing of collagen molecules within the fibrillary structure.

From the folding of the 2D WAXS pattern into the 1D profile (Figure 2B) two diffraction signals were clearly identified: the *meridional* (Me) diffraction peak at q = 2.22 Å^−1^, corresponding to the distance of 2.8 Å between adjacent amino acid residues along the central axis of helical structure, and the *equatorial* (Eq) diffraction peak at q = 0.58 Å^−1^, corresponding to the average apparent distance of 10.8 ± 0.8 Å between the triple helices (typical d-spacing of dry collagen molecules). An additional amorphous-like peak at about q = 1.4 Å^−1^, centered at an average distance of 4.5 Å and related to the distance between collagen skeletons (Sun et al., 2017), was also detected. The Full-Width-at-Half-Maximum (FWHM) of the equatorial peak was extracted from the data (Figure 2C), by Gaussian fitting, in order to obtain information about the crystalline domain of the triple helices lateral packing in the material. Table 3 summarizes these findings.

Table 3WAXS and SAXS data of lateral packing, extent of crystalline domain (FWHM) and fibrillary axial periodicity of type I collagens in thin films obtained by acidic dissolution (AA) and homogenization of collagen acidic solution (OMO), with and without DHT crosslinking treatment.

**Table:** 

**CC_A**						
**Lyophilized commercial collagen**						
		**WAXS**		**SAXS** ***(supramolecular order)***		
	**Lateral d-spacing**	**FWHM(Å**^**−1**^**)**	**Axial periodicity**	**FWHM (Å**^**−1**^**)**	**A (Å**^**−1**^**)**	
NO CROSS	*RAW*	10.8	0.116 ± 0.004	–	–	–
	*OMO*	11.4	0.137 ± 0.004	–	–	–
DHT	*OMO*	11.6	0.119 ± 0.004	–	–	–

**Table:** 

**TYP1CH_A**						
**Chemically extract and lyophilized**						
		**WAXS**		**SAXS** ***(supramolecular order)***		
	**Lateral d-spacing**	**FWHM (Å**^**−1**^**)**	**Axial periodicity**	**FWHM (Å**^**−1**^**)**	**A (Å**^**−1**^**)**	
NO CROSS	*RAW*	10.8	0.098 ± 0.004	60.5	–	–
	*OMO*	11.2	0.100 ± 0.004	61.3	0.0033 ± 0.0005	3.432 ± 0.005
DHT	*OMO*	11.0	0.107 ± 0.004	61.2	0.0039 ± 0.0005	3.737 ± 0.005

**Table:** 

**TYP1CH_B**						
**Chemically extract and hydrated**						
		**WAXS**		**SAXS** ***(supramolecular order)***		
	**Lateral d-spacing**	**FWHM (Å**^**−1**^**)**	**Axial periodicity**	**FWHM(Å**^**−1**^**)**	**A (Å**^**−1**^**)**	
NO CROSS	*AA*	11.0	0.100 ± 0.004	61.8	0.00302 ± 0.0005	5.057 ± 0.005
	*OMO*	11.2	0.100 ± 0.004	61.3	0.00338 ± 0.0005	4.440 ± 0.005
DHT	*AA*	11.0	0.098 ± 0.004	60.7	0.00312 ± 0.0005	5.245 ± 0.005
	*OMO*	11.2	0.098 ± 0.004	61.0	0.00369 ± 0.0005	4.764 ± 0.005

**Table:** 

**TYP1EN_A**						
**Enzymatically extract and lyophilized**						
		**WAXS**		**SAXS** ***(supramolecular order)***		
	**Lateral d-spacing**	**FWHM (Å**^**−1**^**)**	**Axial periodicity**	**FWHM (Å**^**−1**^**)**	**A (Å**^**−1**^**)**	
NO CROSS	*RAW*	10.8	0.101 ± 0.004	–	–	–
	*OMO*	11.6	0.106 ± 0.004	62.3	0.00407 ± 0.0005	3.765 ± 0.005
DHT	*OMO*	11.4	0.088 ± 0.004	61.7	0.00514 ± 0.0005	3.293 ± 0.005

**Table:** 

**TYP1EN_B**						
**Enzymatically extract and hydrated**						
		**WAXS**		**SAXS** ***(supramolecular order)***		
	**Lateral d-spacing**	**FWHM (Å**^**−1**^**)**	**Axial periodicity**	**FWHM (Å**^**−1**^**)**	**A (Å**^**−1**^**)**	
NO CROSS	*AA*	11.4	0.106 ± 0.004	61.5	0.00430 ± 0.0005	3.349 ± 0.005
	*OMO*	11.4	0.109 ± 0.004	–	–	–
DHT	*AA*	11.4	0.104 ± 0.004	60.6	–	–
	*OMO*	11.2	0.108 ± 0.004	–	–	–

**Figure 3:**
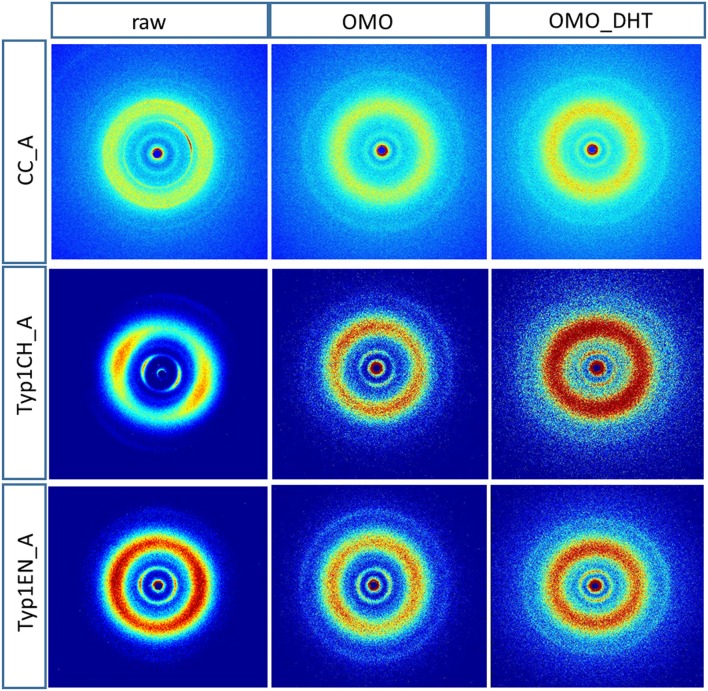
2D WAXS diffraction patterns of A type raw collagen samples and thin films fabricated by homogenization protocol (OMO) and with crosslinking treatment (OMO_DHT).

Figure 3 shows the 2D WAXS patterns collected on all A type raw equine collagens and collagen-based films.

WAXS results can be outlined as:

1. WAXS patterns of CC raw collagen and CC films (first row of Figure 3) show the presence of full rings for meridional and equatorial contributions, while TYP1EN and TYP1CH raw samples have the typical cross fiber diffraction pattern, where both meridional and equatorial reflections are arches oriented along specific directions (preferred orientation).
2. For TYP1EN and TYP1CH films, the preservation of the preferred orientation is dependent on the starting material (ch, en) and crosslinking (DHT). Concerning the effect of fabrication protocols on the material, data suggest that the homogenization process, regardless of the lyophilized (A) or hydrated (B) status of the starting collagen, preserves the preferential orientation of triple helices along the equatorial direction, highlighted by the anisotropic distribution of the diffracted intensity in correspondence of the equatorial diffraction peak (see black arrow, Figure 2A). Conversely, after the DHT crosslinking, the fiber diffraction arches were wider, indicating a reduction in the molecule's spatial orientation, in particular in films obtained by TYP1EN. With regard to the AA treatment, it was observed that it induced a diffracted intensity distributed along a whole ring, indication of a complete loss in the preferred spatial orientation.
3. The extraction treatments do not alter the lateral distance of collagen molecules (WAXS d-spacing 10.8 ± 0.5 Å), nor do it affect the extent of the crystalline domain (lateral molecular organization) at the atomic scale. The mild shift of the equatorial diffraction peak position to higher values, registered for TYP1EN_A, i.e., a wider collagen lateral packing (d-spacing ~11.6 ± 0.5 Å in Table 3) with respect to the corresponding raw values (d-spacing ~10.8 ± 0.5 Å), is probably related to the water incorporation during slurries synthesis, that allows for the formation of an hydration cylinder useful for the molecular structure stabilization.
4. All raw samples possess the same extent of the crystalline domain, as proven by the same FWHM evaluated for the equatorial peak (Table 3).

**Figure 4:**
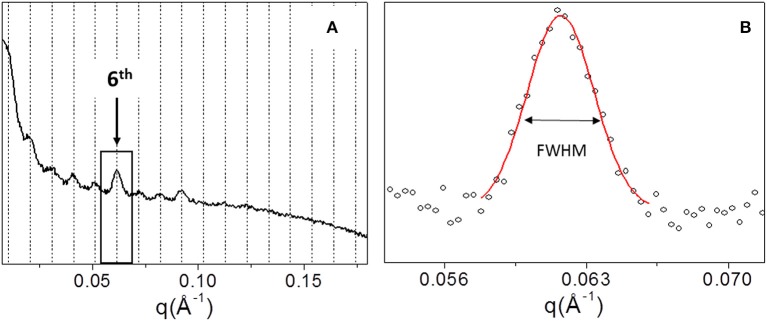
1D SAXS profile of TYP1CH_OMO_A **(A)**. Gaussian fit elaboration and FWHM calculation of the 6th diffraction peak **(B)**.

In order to explore the supramolecular organization, SAXS data were collected for all samples. For example, Figure 4A shows the 1D SAXS profile of TYP1CH OMO_A. The 6th peak of the 1D SAXS profile (see arrow in panel a) was chosen for the analysis of nanocrystalline domain because of the flat background. Fitting the 6th peak with a Gaussian function, the FWHM was determined (see Figure 4B) and reported for each sample in Table 3.

**Figure 5:**
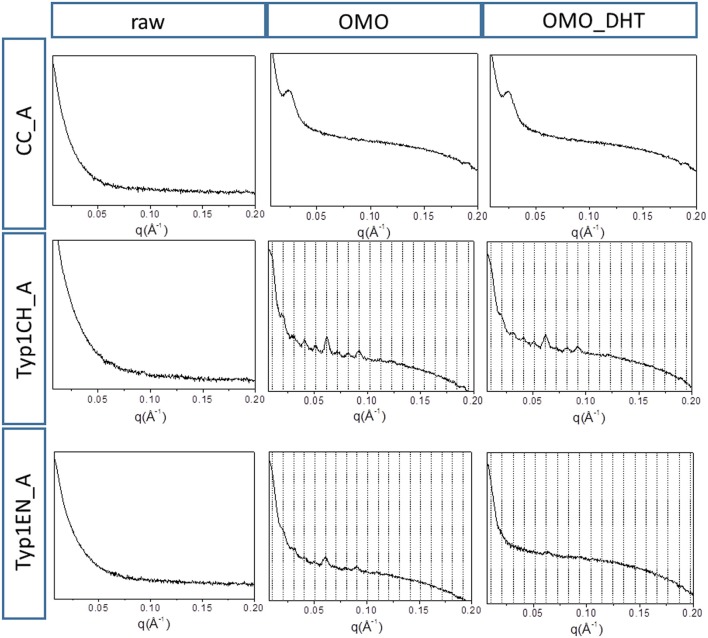
1D SAXS diffraction profiles of A type raw collagen samples and thin films fabricated by homogenization protocol (OMO) and with crosslinking treatment (OMO_DHT).

Figure 5 shows the 1D SAXS patterns collected on all A type raw equine collagens and collagen-based films. SAXS results can be summarized as follows:

- all raw collagen powders do not show any SAXS diffraction peaks at the nanoscale.
- for films, SAXS diffraction peaks were measured in both TYP1EN and TYP1CH, but not in CC, indicating a partial recovery of fibrillary hierarchy at supramolecular scale (particularly striking in TYP1CH).
- an axial periodicity of D = 60.6–62.3 nm was detected in both TYP1CH and TYP1EN films, for whatever processing conditions and crosslinking treatments.
- the homogenization (OMO) appears to increase the FWHM with respect to the other synthesis protocols, indicating the decrease of the nanocrystalline domain in all TYP1 samples.
- a mild decrease of the fibrillary axial periodicity value for the TYP1EN AA and OMO treated (A and B collagens) after DHT crosslinking procedure was observed, probably related to the absence of telopeptides in TYP1EN isoform.
- from a careful comparison of 1D SAXS profiles, we could observe that DHT crosslinking reduces the diffraction signals visibility (peak/background intensity ratio) with respect to the non-crosslinked films while the AA fabrication protocol increases it with respect to the OMO treatment.

Comparing WAXS and SAXS results, we can additionally affirm that:

- For films, comparing the FWHM of the WAXS equatorial peaks in Table 3, it is worth noting that fabrication protocols do not affect the atomic scale structure of the materials whenever the hierarchically organized structure (SAXS signal) is clearly detectable, i.e., for TYP1CH films synthetized from both lyophilized (A) and hydrated (B) collagen.
- Regarding the lyophilized isoform CC_A, where SAXS is not detected, fabrication protocols affect the atomic scale structure. Indeed, an unexpected increase up to FWHM = 0.137 ± 0.004 Å^−1^ in the OMO_ A non-crosslinked film is detected, i.e., increased disorder of collagen molecules, that decreases once again after DHT condensation process (FWHM = 0.119 ± 0.004 Å^−1^).
- TYP1EN_A enzymatically extracted isoform is somehow a mixed situation between CC_A and TYP1CH, where even in the presence of a SAXS pattern, fabrication protocols affect the atomic scale structure. Indeed, the FWHM = 0.106 ± 0.004 Å^−1^ for OMO and non-crosslinked samples decreases down to FWHM = 0.088 ± 0.004 Å^−1^ for OMO and DHT.
- Conversely to the lyophilized A form of TYP1EN, collagen-based films obtained by the hydrated fibers (B) showed almost unchanged FWHM, independently of the processing conditions.

### FT-IR Spectroscopy

FT-IR spectroscopy was performed to analyze CC, TYP1CH and TYP1CH samples in order to evaluate the variation of triple helices numbers in raw samples and thin films due to different processing conditions. Indeed, this technique is commonly used for the characterization of secondary structure of proteins, such as collagen, within biomaterials (Belbachir et al., 2009; Haugh et al., 2009; Bettini et al., 2015, 2017; Bonfrate et al., 2017).

In particular, amide I, the most intense signal of a protein infrared spectrum, located between 1,600 and 1,700 cm^−1^, mainly due to C=O stretching mode, could provide important information about the protein or peptides' secondary structure (Byler and Susi, 1986). In this context, FT-IR was already demonstrated to be a powerful approach to estimate collagen triple helices amount vs. α-helices amount by de-convoluting the amide I band. In fact, the triple helix has a peculiar absorption feature located at about 1,631 cm^−1^, whilst the α-helix is usually centered at about 1,658 cm^−1^ (Goormaghtigh et al., 2006; Petibois et al., 2006; Terzi et al., 2018).

**Figure 6:**
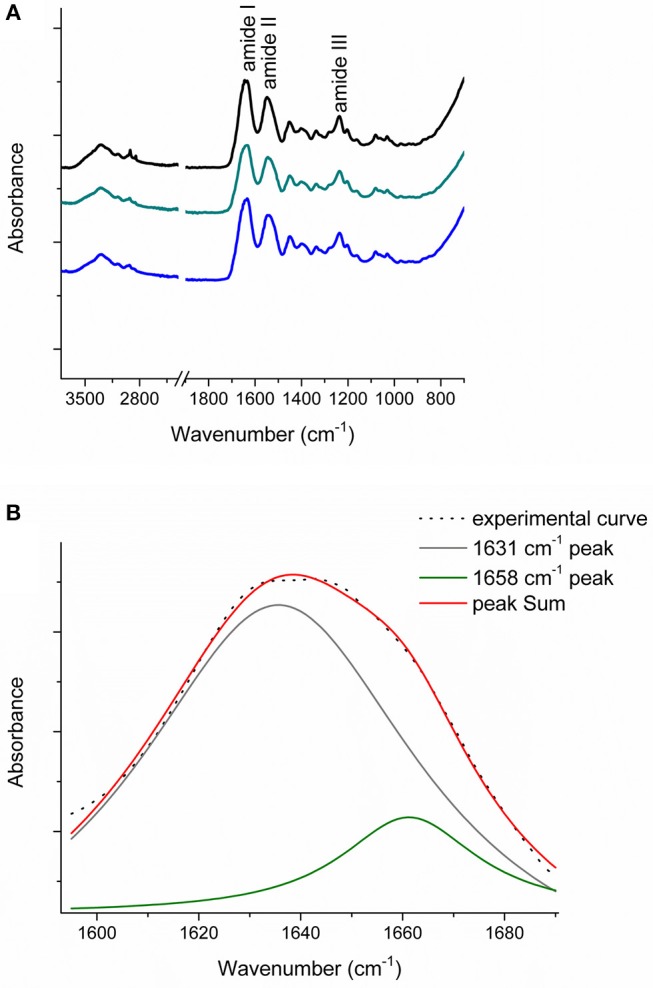
**(A)** FT-IR spectra in the 3,600–700 cm^−1^ range of the raw equine collagens: CC (black), TYP1EN (dark cyan) and TYP1CH (blue); **(B)** Amide I peak de-convolution of α-helix (green line) and triple helix contributes (gray line) elaborated by experimental curve (scatter plot) in CC raw equine collagens.

Figure 6A reports the FT-IR spectra obtained for the raw equine collagens: CC (black line), TYP1CH (blue line) and TYP1EN (dark cyan line). The spectra show the peculiar FT-IR features, in the 3,600–700 cm^−1^ range, of protein samples and, in particular, of type I collagen, being dominated by the amide I (C=O stretching, *ca* 1,642 cm^−1^), amide II (in plane N-H bending, *ca* 1,546 cm^−1^) and amide III (C-N stretching *ca* 1,236 cm^−1^) peaks.

The asymmetrical collagen amide I band was de-convoluted in order to separate the contributions arising from single α-helices (1,658 cm^−1^) from the one related to the triple helix organization (1,631 cm^−1^) and to evaluate the relative amount of triple helices for the investigated raw equine collagens. Figure 6B reports an example the deconvolution carried out on CC.

**Figure 7:**
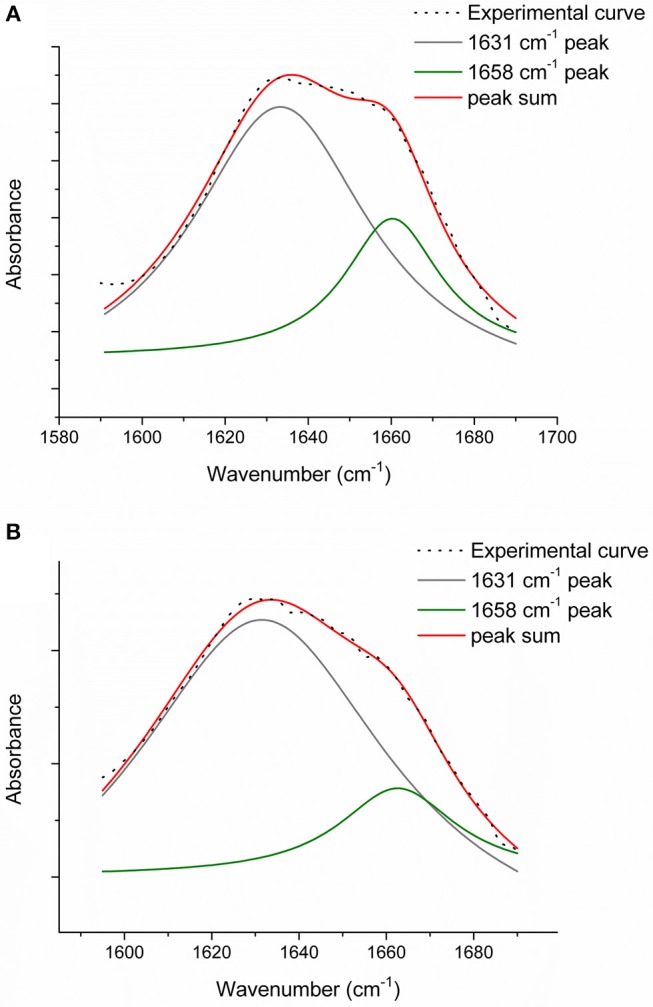
Amide I peak de-convolution of α-helix (green line) and triple helix contributes (gray line) elaborated by experimental curve (scatter plot) in CC collagen samples: not crosslinked **(A)** and DHT **(B)**.

In order to evaluate the relative number of triple helices, depending on both the collagen isoforms and the fabrication protocols, CC, TYP1CH and TYP1EN films were characterized by means of FT-IR spectroscopy and for all the investigated cases the amide I was de-convoluted as previously described. Figure 7 reports the deconvolution carried out on CC film without crosslinking (A) and DHT treatment (B).

The triple helix content (THC) was evaluated as:

(3) $$\begin{array}{l} THC\;\left(\%\right)=100*\frac{\int_{{1590\;cm}^{-1}}^{{1680\;cm}^{-1}}\left(triple\;helix\;curve\right)d\bar{v}}{\int_{{1590\;cm}^{-1}}^{{1680\;cm}^{-1}}\left(amide\;I\;curve\right)d\bar{v}} \end{array}$$

obtaining THC values in percentage, as shown in Table 4. For statistical purposes, each spectrum was collected three times (as the average of 64 scans) and the deconvolution procedure was carried out for each collected spectrum. Then, the triple helix amount (%) was calculated as the average of the three obtained values.

**Table 4:** The relative number of triple helices (%) calculated by FT-IR spectroscopy for the investigated raw samples and films (data are reported as the average of three measurements).

**Sample**				**%THC**
CC	A	RAW		93,7%
		OMO	NO CROSS	81,5%
		OMO	DHT	88,7%
TYP1CH	A	RAW		94,4%
		OMO	NO CROSS	92.3%
		OMO	DHT	91,1%
	B	AA	NO CROSS	80.6%
		AA	DHT	93,2%
		OMO	NO CROSS	82,8%
		OMO	DHT	80,5%
TYP1EN	A	RAW		89,5%
		OMO	NO CROSS	96,1%
		OMO	DHT	76,1%
	B	AA	NO CROSS	83,0%
		OMO	DHT	86,5%
		OMO	NO CROSS	97,4%
		OMO	DHT	77,8%

Regarding CC_A, data reported in Table 4 reveal that the raw sample is characterized by a high number of triple helices of 93.72%. This value is reduced to 81.5% for CC_A films upon the non-crosslinked homogenization process. In contrast, if the film is crosslinked by DHT, the THC value increases to 88.7%. According to previous investigations (Giraud-Guille, 1992), this evidence could be explained with the formation of new chemical bonds (i.e., peptide bonds) among single α-helices that super-organize themselves into triple helices. Moreover, these results are in good agreement with the WAXS analysis previously discussed. Indeed, among the CC_A values, the highest FWHM = 0.137 ± 0.004 Å^−1^ of the CC_A data (Table 3) corresponds to the lowest THC% (Table 4).

Regarding TYP1 raw samples, a higher THC content was found for TYP1CH (94.4%) with respect to TYP1EN (89.5%), correlating with WAXS data which have revealed similar results of FWHM for these two samples. The lower THC value could be ascribed to the enzymatic extraction procedure exploited to obtain the TYP1EN collagen sample, which probably reduces the triple helix configuration.

Regarding TYP1 films without crosslinking, both TYP1CH and TYP1EN reveal, with respect to CC_A, a greater triple helical content. In particular, TYP1EN shows a THC >80% in all the investigated processing methods, reaching 97.4% when OMO films are obtained by hydrated collagen (B) and 96.1% when they are fabricated by lyophilized material (A). This evidence suggests that the homogenization (OMO) process allows collagen α-helix amino acids to easily interact, facilitating the organization in the triple helix configuration. In the same way, TYP1CH film shows 92.3% THC when obtained from lyophilized molecules (A) and OMO treated films, highlighting once again the key role of OMO treatment. It is also worth noting that some unexpected THC% variations were identified in TYP1CH crosslinked by DHT. Indeed, the treatment appears to induce the THC% increase in AA films, according to the cross-linking procedure. Moreover, the DHT treatment does not change the THC amount in OMO treated films, remaining close to 90% (A) and 80% (B). The same is valid for TYP1EN AA films, which show a THC >83%, regardless of the crosslinking. Conversely, unexpected THC values were found in lyophilized and hydrated derived TYP1EN OMO films: indeed, in both cases the crosslinking induces a decrease of about 29% of THC, suggesting a reduction in helical numbers due to the thermal effect. This evidence is particularly clear in TYP1EN OMO_A DHT, in which THC% reaches the lowest value (76.1%) with respect to the other samples, correlating with the lowest FWHM = 0.088 ± 0.004 Å^−1^ obtained by WAXS investigations.

### Mechanical Characterization

**Figure 8:**
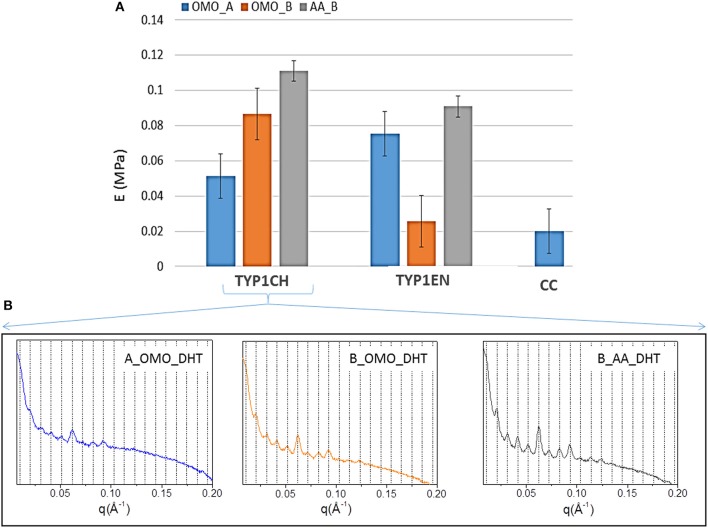
**(A)** Young's moduli of equine collagen-based DHT treated films produced by TYP1CH, TYP1EN and CC isoforms. Histogram directly compares the average moduli for each sample as a function of starting material, crosslinking treatment and processing. Error bars represent the standard deviation. **(B)** 1D SAXS profiles of TYP1CH DHT films obtained from hydrated (collagen B) and lyophilized (collagen A) collagen and treated by homogenization (OMO) and acidic dissolution (AA).

In order to stabilize the collagen structure and allow further manipulation, mechanical characterization of hydrated collagen substrates requires preliminary crosslinking treatments. Therefore, in this study collagen films were subjected to DHT crosslinking, before subsequent tensile tests. The aim was to verify the effect of different collagen isoforms and processing conditions on the collagen stiffness, achieved upon a given crosslinking treatment. DHT crosslinking was particularly selected, since it is a simple method to strengthen the collagen structure without using exogenous chemical agents, and it is commonly performed preliminarily to further increase effective crosslinking methods (e.g., EDAC). The upper part of Table 5 and Figure 8 summarize the results of the elastic Young's modulus (E) extracted from tensile tests (see Methods). As expected, the stiffness of the films is highly affected by the specific collagen and fabrication protocols used. Findings indicate that, after DHT crosslinking, AA films (obtained from both TYP1CH and TYP1EN collagens) are those that show the highest stiffness, compared to OMO films.

**Table 5:** The elastic Young's moduli (E) extracted by tensile tests performed on collagen-based films.

**Sample**				**E (MPa)**
CC	A	OMO	DHT	0.020 ± 0.010
TYP1CH	A	OMO	DHT	0.051 ± 0.010
	B	AA	DHT	0.111 ± 0.010
		OMO	DHT	0.090 ± 0.014
TYP1EN	A	OMO	DHT	0.075 ± 0.010
	B	AA	DHT	0.090 ± 0.004
		OMO	DHT	0.025 ± 0.004

This is particularly evident for TYP1CH collagen, for which significant differences are detected among the various fabrication protocols (AA_B vs. OMO_B, *p* = 0.03; AA_B vs. OMO_A, *p* < 0.0001; OMO_A vs. OMO_B, *p* = 0.003) (see Figure 8). Interestingly, this result correlates with the behavior of the 1D SAXS profiles, reported in the lower part of the figure, which shows an increase of the diffraction signals visibility: OMO_A < OMO_B < AA_B. Furthermore, AA films made of TYP1CH collagen also show the highest THC% increase upon DHT crosslinking (Table 4). With regard to TYP1EN collagen, the AA films show a significantly higher modulus compared to OMO_B films (*p* < 0.0001), while the difference between AA and OMO_A films is not significant (*p* = 0.12). Furthermore, it is interesting to observe that OMO_A films (obtained from lyophilized material) show a higher stiffness for TYP1EN collagen compared to TYP1CH one (*p* = 0.02). Conversely, OMO_B films (obtained from hydrated material) are much stiffer in case of TYP1CH collagen (*p* < 0.0001). However, these significant differences are not detected when considering AA films (*p* = 0.06). Therefore, the collagen extraction protocol seems to affect the film stiffness achieved upon DHT crosslinking, only when the material is subjected to mechanical homogenization. This is somehow consistent with the findings on the THC% content, which denote a very different response of TYP1CH and TYP1EN collagens to DHT crosslinking, following homogenization (with almost no change of the THC% upon DHT treatment for TYP1CH collagen, while significant THC% decrease is found for TYP1EN one). Moreover, regardless of the extraction treatment, films obtained from TYP_ET collagens possess higher E values than CC, when processed by the OMO_A protocol (CC vs. TYP1CH, *p* = 0.011; CC vs. TYP1EN, *p* < 0.0001).

## Discussion

The double aim of the present work was to investigate structural features at the atomic and nano- scales of type I collagen isoforms extracted by equine tendons through different procedures (i.e., chemical and enzymatic extraction) and to assess the impact of different fabrication protocols on structural and mechanical properties of different *collagens*. First, the purity of the collagen isoforms (TYP1CH, TYP1EN, and CC_en) was evaluated using the SDS PAGE technique. The presence of both typical bands of type I collagen, in particular α1(I) of about 130 kDa and α2(I) chains of about 110 kDa, and the detection of the double amount of α1(I) chain with respect to α2(I), highlight the high purity of the collagens.

Crystallographic characterization of raw materials elucidates that the extraction treatments do not alter the lateral packing of collagen molecules (WAXS d-spacing 10.8 ± 0.5 Å), nor affects the extent of crystalline domain (lateral molecular organization) at the atomic scale. Only a mild difference among TYP_ET and CC collagens FWHM was detected, leading to a smaller extent of the crystalline domain in the CC. TYP_ET collagens also showed a preferred orientation of the collagen fibers, which was not detected for CC. Moreover, as assessed by FTIR analysis, raw CC and TYP1CH collagens showed similar triple helices content (THC about 94%), while TYP1EN isoform showed a lower THC (about 89%).

Interestingly, all raw collagen powders did not show any SAXS diffraction peaks at the nanoscale; however, when processed in water solutions to obtain collagen-based films, SAXS diffraction peaks were measured for both TYP1 collagens (ch and en), but not for CC. This indicates, only for TYP1 collagens, a partial recovery of the fibrillary hierarchy at supramolecular scale, upon material hydration. This recovery was especially evident for TYP1CH collagen, while being less pronounced for TYP1EN. Therefore, the collagen extraction protocol seems to affect the capability of the protein to form a fibrillary structure upon hydration, with enzymatically extracted collagen (devoid of C- and N- termini) showing a lower extent of supramolecular hierarchy. In this regard, it is worth recalling that CC collagen is also enzymatically extracted, but its structural features (lower crystalline domain and no preferred orientation) likely make it incapable of partially recovering the fibrillary structure upon hydration. Moreover, CC films also showed a lower THC% than TYP1 films (for similar processing), thus suggesting a relationship between the higher number of triple helices and their supramolecular organization.

Going into further detail, the specific protocols used to fabricate the films (OMO_A, OMO_B and AA_B), as well as the subsequent crosslinking treatment (DHT), were found to affect the collagen structure in different ways, depending on the extent of supramolecular hierarchy. In particular, results from X-ray investigations suggest that the atomic scale structure of the collagen is not affected by the fabrication methods whenever a supramolecular organization is detected, as in the case of TYP1CH. This is like a “shield effect” of the nanoscale architecture to the molecular structure, suggesting that the fabrication treatment primarily affects the fibrils that protect triple helices. Conversely, when the collagen shows a null or less pronounced nano-architecture, as in the case of CC and TYP1EN collagens, the manufacturing protocols affect the atomic scale structure of the material.

Combined results from FT-IR and X-rays investigations performed on CC collagen films showed that the homogenization process (OMO) caused a decrease of both the triple helical amount and the extent of crystalline domain. However, subsequent DHT crosslinking was found to increase the crystalline domain and the triple helical amount. These findings thus suggested that mechanical homogenization of collagen, in the absence of a nano-architecture, could induce the fragmentation of the collagen molecules. However, the molecules are then able to reorganize themselves during DHT treatment in an enhanced lateral organization, due to the formation of new bonds (i.e., crosslinks) among α helices. These bonds occur within the triple helical structure of CC collagen, which has not a supramolecular organization and has lost its N- and C- termini.

With regard to TYP1EN collagen, which conversely shows a certain extent of supramolecular hierarchy, the extent of the crystalline domain remained approximately unchanged among non-crosslinked films, independently of the fabrication method (OMO_A, OMO_B, and AA_B) (Table 3). Nonetheless, the THC% (Table 4) was affected by the specific protocol used, with higher THC values (about 96%) reached upon homogenization (OMO_A and OMO_B) compared to the acetic acid treatment (AA_B) (about 83%). These findings may be ascribed to the fact that, also in the presence of a supramolecular structure, the homogenization may induce the fragmentation of the collagen molecules into smaller triple helices (high THC%). This evidence is also in accordance with results previously observed in bovine-dermis derived collagens (Terzi et al., 2018). However, following DHT crosslinking, the THC% was found to lower down to 76–77% for both OMO_A and OMO_B films, while being slightly increased (up to 86%) for AA_B one. Crosslinked OMO_A films were particularly the ones showing the minimum THC% (76.1%) and the maximum extent of crystalline domain (FWHM = 0.088 ± 0.004 Å^−1^). We thus suppose that the remarkable triple helical content reduction, detected for homogenized samples following DHT crosslinking, may be directly related to the concurrent thermal denaturation of collagen occurring during DHT treatment, whereas the increased order is related to the formation of new bonds among α-helices, as already observed for CC films. The high sensitivity of TYP1EN collagen to thermal denaturation might be due to the enzymatic extraction procedure, which cuts the C- and N- termini and likely makes the collagen molecule less stable. Moreover, a mild decrease of fibrillary axial periodicity upon TYP1EN after DHT crosslinking procedure was also observed. Indeed, as already known from literature, the application of the dehydrothermal treatment for more than 24 h can induce the reduction of D-spacing values (fibrillary axial periodicity) (Cameron et al., 2002).

As for TYP1CH collagen, which shows a higher level of supramolecular organization than TYP1EN one, the extent of the crystalline domain is almost unaffected by the fabrication method (OMO_A, OMO_B, and AA_B) (Table 3). Similar to that observed for TYP1EN collagen, the THC% (Table 4) was affected by the specific protocol used: in particular, higher THC values (about 92%) were reached upon homogenization of lyophilized fibers (OMO_A), while hydrated fibers showed comparable THC% values for both acetic acid and homogenization treatments (AA_B and OMO_B) (about 80–83%). Furthermore, it is worth noting that, for OMO_A and OMO_B films, the THC% was only slightly reduced following DHT crosslinking (down to 91 and 80%, respectively), thus suggesting that chemically extracted collagen is much more stable with respect to thermal denaturation than enzymatically extracted collagen. On the contrary, for AA_B films the THC% was largely increased upon DHT crosslinking, up to 93%.

In accordance with the increased crystalline domain and supramolecular structure of TYP1 films with respect to CC ones, mechanical tests highlighted a much higher stiffness of TYP1 films upon DHT crosslinking (Figure 8). Moreover, when comparing TYP1CH and TYP1EN collagens, AA_B films were found to show the maximum elastic modulus, which was approximately the same for both collagens. This stiffness enhancement upon acidic (AA) treatment was also consistent with previous observations on bovine substrates (Terzi et al., 2018).

On the contrary, the homogenization process was found to have different effects on the stiffness achieved upon DHT crosslinking. Indeed, OMO_B films (obtained from hydrated fibers) and OMO_A films (obtained from lyophilized fibers) were found to be stiffer for TYP1CH and TYP1EN collagen, respectively. Such differences between the two collagens may be related to their different sensitivity to crosslinking and thermal denaturation, which may both occur upon DHT treatment (Tung et al., 2009). In particular, crosslinking and denaturation have opposite effects on stiffness, inducing an increase and a decrease of stiffness, respectively. Our findings seem to suggest that enzymatically extracted collagen (TYP1EN) is less prone to supramolecular organization and more susceptible to denaturation, compared to the chemically extracted one (TYP1CH). However, it may also show an increased order following DHT treatment (e.g., OMO_A), due to the formation of new bonds among α-helices. Therefore, the stiffness achieved upon DHT crosslinking not only depends on the extent of fibrillary assembly (as clearly shown for TYP1CH collagen, Figure 8B), but also on the counteracting crosslinking and denaturation involved in the process.

## Conclusions

Wide Angle X-rays Scattering technique reveals a preferential orientation of molecules in raw TYP1 collagens compared to CC, but the extent of the crystalline domains is not affected by the extraction and purification treatment (chemical vs. enzymatic).

On the contrary, film fabrication protocols affect the structural properties of the material in different ways, depending on the extent of supramolecular hierarchy, which is in turn related to the extraction protocols.

In particular, for chemically extracted TYP1CH collagen films, a stable nano-architecture is observed, which basically acts as a shield, protecting the molecular structure from potential damage that may occur during mechanical processing (by homogenization) and/or DHT crosslinking (i.e., thermal denaturation). This shielding effect is much less pronounced in enzymatically extracted TYP1EN collagen films, for which SAXS analysis reveals a less marked recovery of supramolecular structure after material hydration. Indeed, combined WAXS and FTIR analyses suggest that, for TYP1EN collagen, the shield effect is able to protect the collagen molecules from mechanical fragmentation, but not from the partial denaturation induced by DHT condensation. However, upon DHT crosslinking, the collagen molecules can also reorganize themselves in an enhanced lateral organization, thanks to the formation of new bonds (i.e., crosslinks). As for the collagen without a supramolecular organization (i.e., CC), mechanical homogenization is found to induce the fragmentation of the collagen molecules (no shield effect). Nonetheless, also in this case, the collagen molecules are able to reorganize themselves in an enhanced lateral organization during DHT crosslinking.

Finally, with regard to the mechanical stiffness of collagen-based films, our results suggest that the stiffness achieved upon DHT crosslinking is due to the combination of various interacting variables, including the extent of supramolecular assembly, as well as the crosslinking and the denaturation yielded by DHT treatment. In particular, AA films show the highest stiffness, especially for TYP1CH collagen, thus confirming the results that have already been observed on bovine collagen substrates and also correlating with the increase of the SAXS signal visibility on AA films, with respect to the OMO-treated ones.

In brief, this work sheds light on the influence of the extraction processes on collagen's secondary and supramolecular structure and on the peculiar response of the material to different processing conditions. It may thus be very useful in the design of optimized collagen-based scaffolds for tissue engineering applications.

## Data Availability

The datasets generated for this study are available on request to the corresponding author.

## Author Contributions

LC and MN extracted and purified TYP1EN/TYP1CH collagens. AT synthetized collagen-based films. NG performed the SDS page analysis. AT, TS, DA, and CG realized WAXS and SAXS experiments on raw collagens and films at XMI-L@b. LD developed the restoring algorithm. AT performed mechanical tests under the supervision of LS, MM, and AS. SB performed FT-IR experiments under the supervision of LV. AT, TS, DA, NG, LS, MM, SB, LV, AS, and CG discussed the results and wrote the paper in close collaboration with all the authors. All authors have approved the final version of the manuscript.

### Conflict of Interest Statement

The authors declare that the research was conducted in the absence of any commercial or financial relationships that could be construed as a potential conflict of interest.
