# Supplementary material for: Investigations of Processing–Induced Structural Changes in Horse Type-I Collagen at Sub and Supramolecular Levels
Source: Front Bioeng Biotechnol. 2019 Aug 26;7:203. doi: 10.3389/fbioe.2019.00203 (PMC6736615; doi:10.3389/fbioe.2019.00203)
Supplement: Supplementary file 1 [file Data_Sheet_1.PDF]

Supplementary Material

Investigating horse type-I collagen structural properties as a function of processing at sub and supramolecular level

A.Terzi[1], N. Gallo[2], S. Bettini[2], T. Sibillano[1], D. Altamura[1], L. Campa[4], M.L. Natali[4], L. Salvatore[2], M. Madaghiele[2], L. De Caro[1], L. Valli[3], A. Sannino[2] & C. Giannini [1,*]

[1] *Institute of Crystallography (IC), National Research Council, Bari, Italy*

[2] *Department of Engineering for Innovation, University of Salento, Lecce, Italy*

[3] *Department of Biological and Environmental Sciences and Technologies, University of Salento, Lecce, Italy*

[4] *Typeone Srl, Lecce, Italy*

*** Correspondence:**
Dr. Cinzia Giannini
cinzia.giannini@ic.cnr.it

1 Supplementary Figures and Tables

1.1 Supplementary Figures

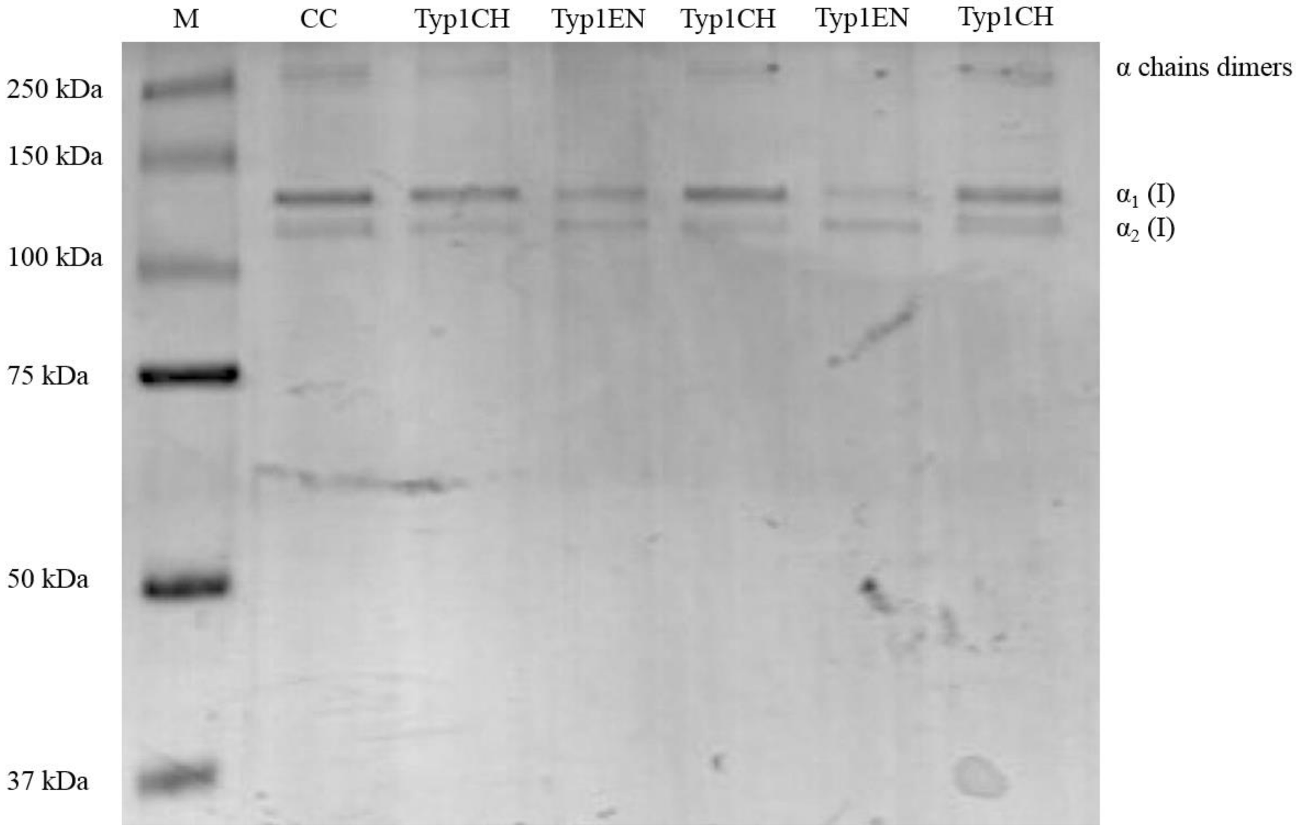

Supplementary figure 1. SDS PAGE of the full length collagen gel: marker M (broad range 37-250kDa), CC (commercial collagen) used as control for the purity assessment; TYP1CH and TYP1EN collagens in triplicate and duplicate respectively.
